# *Dictyostelium* Differentiation-Inducing Factor 1 Promotes Glucose Uptake via Direct Inhibition of Mitochondrial Malate Dehydrogenase in Mouse 3T3-L1 Cells

**DOI:** 10.3390/ijms25031889

**Published:** 2024-02-04

**Authors:** Yuzuru Kubohara, Yuko Fukunaga, Ayako Shigenaga, Haruhisa Kikuchi

**Affiliations:** 1 Laboratory of Health and Life Science, Graduate School of Health and Sports Science, Juntendo University, Inzai 270-1695, Japan; 2 Department of Animal Risk Management, Faculty of Risk and Crisis Management, Chiba Institute of Science, Choshi 288-0025, Japan; yfukunaga@cis.ac.jp; 3 Institute of Health and Sports Science & Medicine, Juntendo University, Inzai 270-1695, Japan; ayamatsu@juntendo.ac.jp; 4 Division of Natural Medicines, Faculty of Pharmacy, Keio University, Tokyo 105-8512, Japan; halkiku@keio.jp

**Keywords:** *Dictyostelium discoideum*, DIF-1, obesity, diabetes, MDH2, AMPK

## Abstract

Differentiation-inducing factor 1 (DIF-1), found in *Dictyostelium discoideum*, has antiproliferative and glucose-uptake-promoting activities in mammalian cells. DIF-1 is a potential lead for the development of antitumor and/or antiobesity/antidiabetes drugs, but the mechanisms underlying its actions have not been fully elucidated. In this study, we searched for target molecules of DIF-1 that mediate the actions of DIF-1 in mammalian cells by identifying DIF-1-binding proteins in human cervical cancer HeLa cells and mouse 3T3-L1 fibroblast cells using affinity chromatography and liquid chromatography–tandem mass spectrometry and found mitochondrial malate dehydrogenase (MDH2) to be a DIF-1-binding protein in both cell lines. Since DIF-1 has been shown to directly inhibit MDH2 activity, we compared the effects of DIF-1 and the MDH2 inhibitor LW6 on the growth of HeLa and 3T3-L1 cells and on glucose uptake in confluent 3T3-L1 cells in vitro. In both HeLa and 3T3-L1 cells, DIF-1 at 10–40 μM dose-dependently suppressed growth, whereas LW6 at 20 μM, but not at 2–10 μM, significantly suppressed growth in these cells. In confluent 3T3-L1 cells, DIF-1 at 10–40 μM significantly promoted glucose uptake, with the strongest effect at 20 μM DIF-1, whereas LW6 at 2–20 μM significantly promoted glucose uptake, with the strongest effect at 10 μM LW6. Western blot analyses showed that LW6 (10 μM) and DIF-1 (20 μM) phosphorylated and, thus, activated AMP kinase in 3T3-L1 cells. Our results suggest that MDH2 inhibition can suppress cell growth and promote glucose uptake in the cells, but appears to promote glucose uptake more strongly than it suppresses cell growth. Thus, DIF-1 may promote glucose uptake, at least in part, via direct inhibition of MDH2 and a subsequent activation of AMP kinase in 3T3-L1 cells.

## 1. Introduction

**Figure 1:**
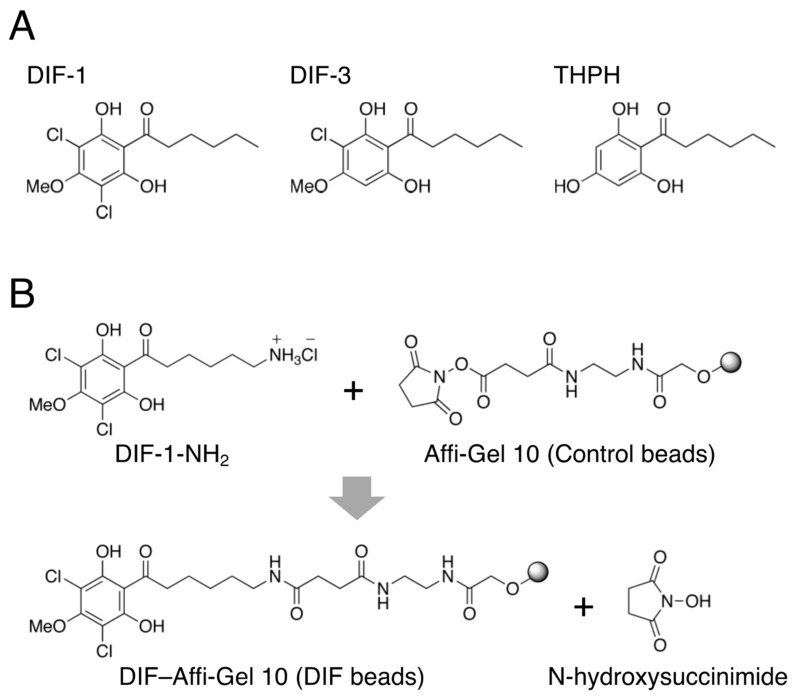
(**A**) Chemical structures of DIF-1, DIF-3, and THPH (a nonbioactive derivative of DIF-1). DIF-1, 1-(3,5-dichloro-2,6-dihydroxy-4-methoxyphenyl)hexan-1-one; DIF-3, 1-(3-chloro-2,6-dihydroxy-4-methoxyphenyl)hexan-1-one; THPH, 1-(2,4,6-trihydroxyphenyl)hexan-1-one. (**B**) Schematic diagram of the coupling reaction between Affi-Gel 10 resin and DIF-1-NH_2_ [6-amino-1-(3,5-dichloro-2,6-dihydroxy-4-methoxyphenyl)hexan-1-one hydrochloride] to make DIF beads [18].

Differentiation-inducing factor (DIF)-1 and DIF-3 (Figure 1A), which are chlorinated alkylphenones, were originally identified as inducers of stalk cell differentiation in the cellular slime mold *Dictyostelium discoideum* [1,2,3,4]. Later, DIF-1, DIF-3, and their derivatives were found to have antiproliferative and antimetastatic activities in mammalian tumor cells in vitro and in vivo [5,6,7,8,9,10,11,12,13]. Notably, DIF-1 and DIF-3 can function as mitochondrial uncouplers in mammalian cells [6,8]. In 2007, we reported that DIF-1 can also promote glucose uptake in mammalian cells, such as the fibroblast and adipocyte states of mouse 3T3-L1 cells, which are used to study diabetes, obesity, and related disorders [14]. DIF-1 promotes glucose uptake by inducing translocation of glucose transporter 1 (GLUT1) from intracellular vesicles to the plasma membrane, at least in part via the mitochondria- and 5′-AMP-activated kinase (AMPK)-dependent and 3′,5′-cyclic adenosine monophosphate (cAMP)-dependent pathways [15,16]. Oral administration of DIF-1 decreases blood glucose levels in streptozotocin-treated diabetic rats [17]. These findings suggest that DIF-1 and its derivatives may have therapeutic potential for the treatment of obesity, diabetes, or both. However, the full picture of the mechanisms underlying the actions of DIFs is not clear and, in particular, the target molecules of DIFs that mediate their actions remain unknown.

As for potential pharmacological target molecules of DIF-1 and DIF-3 in mammalian cells, DIFs have been shown to directly inhibit PDE1 [19], a calmodulin-dependent phosphodiesterase that degrades cAMP and cyclic guanosine monophosphate (cGMP) [20,21,22], and might thereby exhibit anticancer activities [16,19,22]. A previous study identified mitochondrial malate dehydrogenase, known as malate dehydrogenase 2 (MDH2), as a DIF-1-binding protein (DBP) in human cervical cancer HeLa cells and further showed that DIF-1 inhibits MDH2 activity in vitro, but not that of MDH1 (cytoplasmic MDH), whereas DIF-3 affects neither MDH1 nor MDH2 activity [23].

In this study, to elucidate the mechanisms underlying the glucose-uptake-promoting effect of DIF-1, we attempted to identify the pharmacological target molecules of DIF-1 in 3T3-L1 fibroblasts, using HeLa cells as a reference. We performed affinity chromatography using DIF beads (Figure 1B) and liquid chromatography–tandem mass spectrometry (LC–MS/MS). We show here that MDH2 is a DBP in both 3T3-L1 and HeLa cells, and that the MDH2 inhibitor LW6 promotes glucose uptake similarly to DIF-1 in confluent 3T3-L1 cells, suggesting that DIF-1 promotes glucose uptake, at least in part by inhibiting MDH2 in these cells.

## 2. Results

### 2.1. Effects of DIF-1, DIF-3, and THPH on the Growth of HeLa and 3T3-L1 Cells and Glucose Uptake in 3T3-L1 Cells

**Figure 2:**
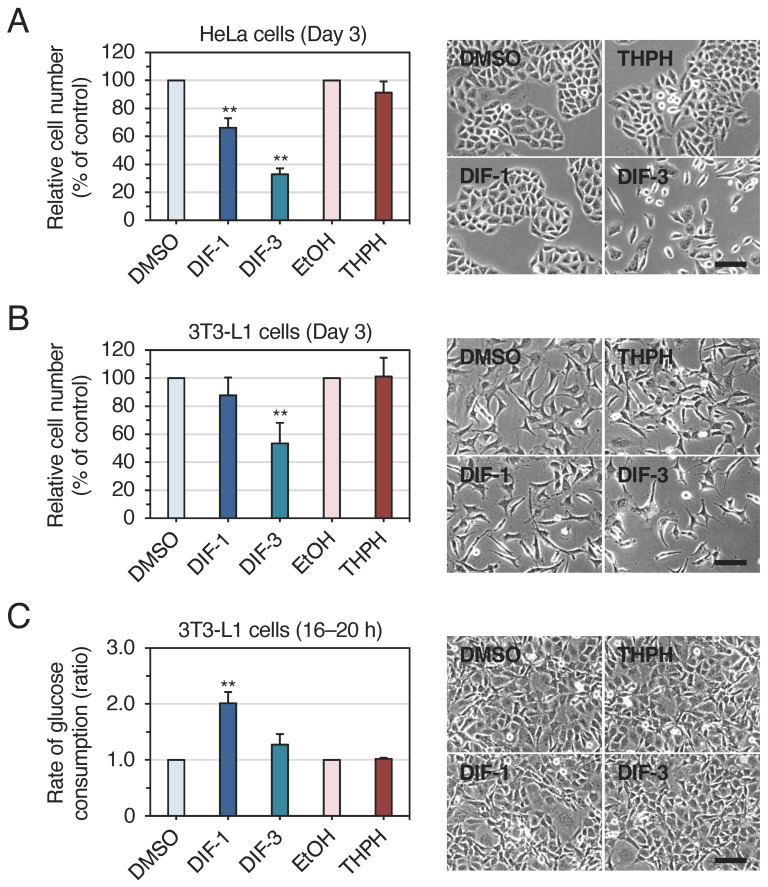
(**A**,**B**) Effects of DIF-1, DIF-3, and THPH on the growth of HeLa and 3T3-L1 cells. Cells were incubated for 3 days in the presence of 0.1% dimethylsulfoxide (DMSO) or ethanol (EtOH) (vehicles) or 20 μM of DIF-1, DIF-3, or THPH, and the relative cell number was determined. The data are mean ± SD of five (**A**) or four (**B**) independent experiments. ** *p* < 0.01 versus control (by *t*-test). Representative photos of the cells after incubation with the indicated compounds are also shown. Bars: 100 μm. (**C**) Effects of DIF-1, DIF-3, and THPH on glucose uptake in 3T3-L1 cells. Confluent 3T3-L1 cells were incubated for 16–20 h in the presence of 0.1% DMSO or EtOH (vehicles) or 20 μM of DIF-1, DIF-3, or THPH, and the rate of glucose consumption was assessed. The data are mean ± SD of three independent experiments. ** *p* < 0.01 versus control (by *t*-test). Representative photos of the cells after 17 h incubation with the indicated compounds are also shown. All the cells look healthy. Bar: 100 μm.

We first compared the effects of DIF-1, DIF-3, and THPH (a nonbioactive derivative of DIF-1) at 20 μM on the growth of HeLa and 3T3-L1 cells in vitro (Figure 2A,B). DIF-1 significantly suppressed the growth of HeLa cells (Figure 2A), and DIF-3 significantly suppressed the growth of HeLa and 3T3-L1 cells (Figure 2A,B), whereas THPH did not affect the growth of either cell type, indicating that DIF-3 has a stronger antiproliferative activity than DIF-1 in mammalian cells and that THPH is a nonbioactive analog of DIFs. These results are largely consistent with previous studies of DIFs in various tumor cells [5,11].

We then compared the effects of DIF-1, DIF-3, and THPH at 20 μM on glucose uptake in confluent 3T3-L1 cells in vitro (Figure 2C). DIF-1 promoted glucose uptake by about 2-fold, whereas DIF-3 might promote it slightly but not significantly, and THPH did not affect it in these cells, indicating that DIF-1 has a stronger glucose-uptake-promoting activity than DIF-3 and that THPH is nonbioactive. Note that none of DIF-1, DIF-3, or THPH affected cell morphology. All these results agree well with our previous report [14].

### 2.2. Identification of DBPs in HeLa and 3T3-L1 Cells

**Figure 3:**
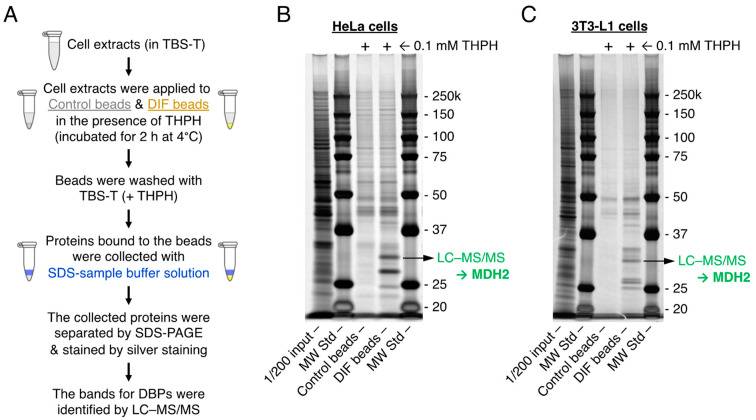
(**A**) Flow diagram for the identification of DIF-binding proteins (DBPs) in HeLa and 3T3-L1 cells. Cells were lysed in 0.05% Tween 20 in Tris-buffered saline (TBS-T) and incubated with control beads or DIF beads in the presence of 0.1 mM THPH to reduce nonspecific protein binding to the beads. The beads were washed several times with TBS-T containing THPH, and the bound proteins were eluted with an SDS-sample buffer. The affinity-purified proteins were separated by SDS-PAGE and stained with silver staining. The proteins that specifically bound to the DIF beads were identified by using LC–MS/MS. (**B**,**C**) Photos of the silver-stained SDS-PAGE gels. Total cell extracts (1/200 input), the proteins bound to control and DIF beads, and molecular weight standards (MW Std) were subjected to SDS-PAGE, and the resulting gel was stained. The protein bands present in the DIF beads lane but not in the control beads lane were collected, and one of the bands was identified as MDH2.

We first attempted to identify DBPs in HeLa cells (Figure 3A,B). We collected cell protein with a mild nonionic detergent (0.05% Tween 20) solution, and performed affinity chromatography in the presence of 0.1 mM THPH (a nonbioactive analog of DIF-1) to reduce nonspecific protein binding to the DIF beads. Affinity chromatography revealed several candidates for DBPs (Figure 3B), and subsequent LC–MS/MS identified one DBP as MDH2. This result is consistent with a previous report in which MDH2 was identified as a DBP in HeLa cells by affinity chromatography (using DIF-conjugated beads different from the beads shown in Figure 1B) and LC–MS/MS and in which DIF-1 was also shown to be a direct inhibitor of MDH2 [23]. Similarly, we identified MDH2 as a DBP in 3T3-L1 cells in the same way (Figure 3C).

Note that several other DBPs were also identified in HeLa and 3T3-L1 cells (Figure 3B,C); we are currently analyzing whether these DBPs are bona fide target molecules that mediate the actions of DIF-1 and its derivatives.

### 2.3. Effects of an MDH2 Inhibitor on the Growth of HeLa and 3T3-L1 Cells and on Glucose Uptake in 3T3-L1 Cells

**Figure 4:**
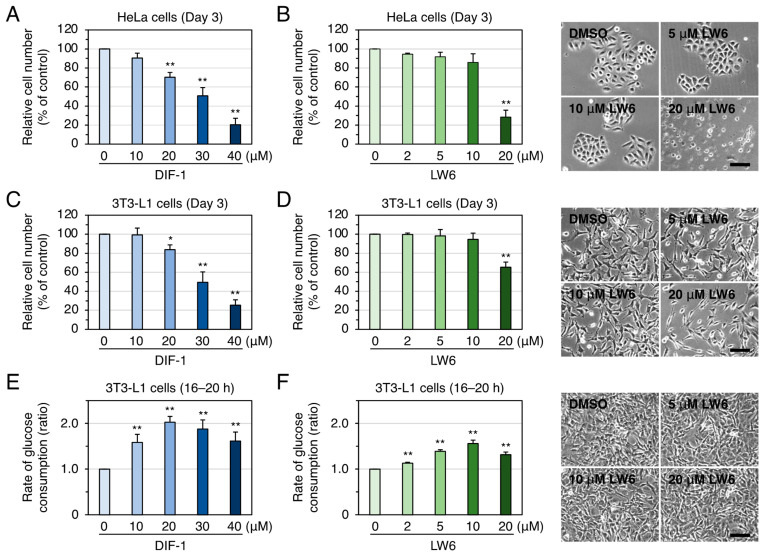
(**A**–**D**) Comparison of the effects of DIF-1 and LW6 (an MDH2 inhibitor) on the growth of HeLa and 3T3-L1 cells. Cells were incubated for 3 days in the presence of 0.2% DMSO (vehicle) or the indicated concentrations of DIF-1 or LW6, and then the relative cell number was determined. The data are the mean ± SD of four (**A**) or three (**B**–**D**) independent experiments. * *p* < 0.05, ** *p* < 0.01 versus control (by *t*-test). Representative photos of the cells after incubation with the indicated concentrations of LW6 are also shown. Bars: 100 μm. (**E**,**F**) Comparison of the effects of DIF-1 and LW6 on glucose uptake in 3T3-L1 cells. Confluent 3T3-L1 cells were incubated for 16–20 h in the presence of 0.2% DMSO (vehicle) or the indicated concentrations of DIF-1 or LW6, and the rate of glucose consumption was assessed. The data are the mean ± SD of four (**E**) or three (**F**) independent experiments. ** *p* < 0.01 versus control (by *t*-test). Representative photos of the cells after 17 h incubation with the indicated concentrations of LW6 are also shown. All the cells look healthy. Bar: 100 μm.

To ascertain whether MDH2 can be a pharmacological target of DIF-1, we needed to determine whether MDH2 inhibition by DIF-1 inhibits cell proliferation and/or promotes glucose uptake, or has no effect on these functions. We thus compared the effects of DIF-1 and the MDH2 inhibitor LW6 [24,25,26,27,28] on the growth of HeLa cells and 3T3-L1 cells in vitro (Figure 4A–D). In both HeLa and 3T3-L1 cells, DIF-1 at 10–40 μM dose-dependently suppressed cell growth (Figure 4A,C), as described previously [16]. LW6 at 20 μM suppressed the growth of these cells but not significantly at 2–10 μM (Figure 4B,D); 20 μM LW6 was rather toxic to HeLa cells, with the cell morphology appearing abnormal (Figure 4B).

We then compared the effects of DIF-1 and LW6 on glucose uptake in confluent 3T3-L1 cells in vitro (Figure 4E,F). DIF-1 at 10–40 μM significantly promoted glucose uptake, with the strongest effect at 20 μM DIF-1 (Figure 4E), as described previously [16]. LW6 at 2–20 μM promoted glucose uptake, with the strongest effect at 10 μM LW6. At up to 20 μM, LW6 was not toxic in terms of cell morphology (Figure 4F).

These results strongly suggest that DIF-1 promotes glucose uptake, at least in part via inhibition of MDH2 in 3T3-L1 cells, and that growth suppression by DIF-1 may involve MDH2 inhibition, but its involvement is small.

### 2.4. Combinatorial Effects of LW6, Dinitrophenol, and 8-bromo-cAMP on Glucose Uptake in 3T3-L1 Cells

**Figure 5:**
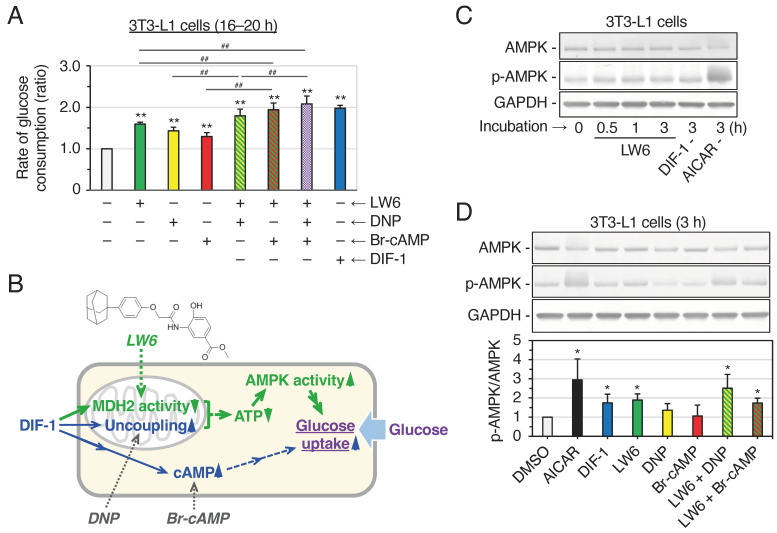
(**A**) Combinatorial effects of an MDH2 inhibitor (LW6), a mitochondrial uncoupler (dinitrophenol; DNP), and a membrane-permeable cAMP analog (8-bromo-cAMP; Br-cAMP) on glucose uptake in 3T3-L1 cells compared to that of DIF-1. Cells were incubated for 16–20 h in the presence of 0.2% DMSO (vehicle) and 10 μM LW6, 0.1 mM DNP, and/or 0.3 mM Br-cAMP, or 20 μM DIF-1, and the rate of glucose consumption was assessed. The data are the mean ± SD of six independent experiments. ** *p* < 0.01 versus control (by *t*-test); ^##^ *p* < 0.01 (by ANOVA). (**B**) Proposed scheme for the action of DIF-1 in 3T3-L1 cells. DIF-1 promotes glucose uptake by uncoupling mitochondria [14,15], increasing intracellular cAMP levels [16], and inhibiting MDH2 (this study). Uncoupling mitochondria and inhibiting MDH2 would decrease cellular ATP levels and activate AMPK, which would promote glucose uptake [15]. MDH2 inhibitor LW6 (with the indicated chemical structure), mitochondrial uncoupler DNP, and membrane-permeable cAMP analog Br-cAMP can mimic the action of DIF-1. The pathways suggested for the first time in this study are shown in green, except that the effects of DIF-1 and LW6 on cellular ATP levels have been shown in other cells [23,25]. (**C**) Effects of LW6 on AMPK activity in 3T3-L1 cells. Cells were incubated for the indicated time periods in the presence of 10 μM LW6, 20 μM DIF-1, or 0.2 mM 5-aminoimidazole-4-carboxamide-1-β-D-ribofuranoside (AICAR), and cell lysates were assessed by Western blotting for AMPK, phospho-AMPK (p-AMPK), and glyceraldehyde 3-phosphate dehydrogenase (GAPDH), an internal control protein. (**D**) Effects of LW6, DNP, and/or Br-cAMP on AMPK activity in 3T3-L1 cells. The cells were incubated for 3 h in the presence of 0.2% DMSO (vehicle), 0.2 mM AICAR, 20 μM DIF-1, 10 μM LW6, 0.1 mM DNP, and/or 0.3 mM Br-cAMP. Cell lysates were assessed by Western blotting for AMPK, p-AMPK, and GAPDH. The graph shows the ratio of p-AMPK/AMPK of each sample to that of the DMSO control; the means ± SD of four independent experiments are presented. * *p* < 0.01 versus DMSO control (by *t*-test).

We have previously shown that DIF-1 promotes glucose uptake, at least in part by increasing intracellular cAMP levels and uncoupling mitochondria in 3T3-L1 cells [15,16]. To elucidate the relationship between MDH2, mitochondrial uncoupling, and intracellular cAMP in the action of DIF-1, we examined the effects of LW6, dinitrophenol (DNP; a mitochondrial uncoupler), 8-bromo-cAMP (Br-cAMP; a membrane-permeable analog of cAMP), or their combinations on glucose uptake in confluent 3T3-L1 cells in vitro (Figure 5A). LW6 (10 μM), DNP (0.1 mM), and Br-cAMP (0.3 mM) significantly promoted glucose uptake in the cells. LW6 in combination with DNP or Br-cAMP promoted it more strongly than each alone, and LW6 in combination with DNP and Br-cAMP promoted it as strongly as DIF-1 (20 μM). These results suggest that DIF-1 promotes glucose uptake by directly inhibiting MDH2, uncoupling mitochondria, and increasing intracellular cAMP levels in 3T3-L1 cells (Figure 5B).

### 2.5. Effects of LW6, DNP, and Br-cAMP on AMPK Activity in 3T3-L1 Cells

Activation (phosphorylation) of AMPK induces translocation of glucose transporter 1 (GLUT1) from intracellular vesicles to the plasma membrane and promotes glucose uptake in various cells [29,30,31,32]. DIF-1 has also been shown to promote glucose uptake, at least in part via the AMPK-dependent pathway in 3T3-L1 cells [15] (Figure 5B). To clarify the relationship between mitochondrial uncoupling, MDH2 inhibition, increase in intracellular cAMP levels, and AMPK activation in DIF-1-promoted glucose uptake (Figure 5B), we examined the effects of LW6 (10 μM), DNP (0.1 mM), Br-cAMP (0.3 mM), or their combinations on AMPK activity and compared their effects with those of DIF-1 (20 μM) and 5-aminoimidazole-4-carboxamide-1-β-D-ribofuranoside (AICAR; 0.2 mM), an activator of AMPK [33,34], by Western blotting (Figure 5C,D). As expected, AICAR greatly activated (phosphorylated) AMPK at 3 h of incubation, and LW6 and DIF-1 significantly activated AMPK, whereas Br-cAMP did not significantly affect AMPK activity. Furthermore, DNP tended to activate AMPK, and LW6 and DNP tended to show an additive effect on AMPK activation. These results support the schematic diagram in Figure 5B, which illustrates the mechanism of DIF-1 action.

## 3. Discussion

### 3.1. DIF-1 and DIF-3 as Leads for the Development of Anticancer and/or Antidiabetic Drugs

Diabetes is a chronic disease in metabolic disorders, and its pathological features are impaired insulin secretion or biological dysfunction, or both, which causes elevated blood sugar levels, whereas obesity is a high-risk factor for diabetes [35,36,37]. There are currently several types of oral hypoglycemic drugs available, including insulin (oral delivery), insulin secretagogues (e.g., dipeptidyl peptidase-4 inhibitors, glucagon-like peptide-1 receptor agonists, and gliclazide), and noninsulin secretagogues (e.g., metformin and thiazolidine) [38,39,40,41]. However, there are cases in which these drugs do not have sufficient therapeutic effects, and the side effects induced by the drugs can be a serious problem [39,40]. Therefore, the development of new drugs for diabetes treatment is still attracting attention [39,42].

DIF-1 and DIF-3 (Figure 1A) were originally isolated as stalk cell differentiation-inducing factors in *D. discoideum* [1,2]. DIF-3 is the initial product in the process of DIF-1 breakdown and is much less active in inducing stalk-cell differentiation [3,4,43,44]. DIFs are thought to penetrate the cell membrane due to their chemical structures and solubility in both hexane and water [3]. Regarding their potential in anticancer therapy, note that DIF-3 has stronger antiproliferative activity than DIF-1 in several mammalian tumor cell lines tested so far [5,6,11], as consistently demonstrated in HeLa cells in the present study (Figure 2A).

Since 2007, we have shown that DIF-1 has strong glucose-uptake-promoting activity in mammalian cells in vitro and possibly in vivo [11,14,15,16,17], but DIF-3 hardly promotes glucose uptake in vitro in confluent 3T3-L1 cells [14], which agrees well with the present data (Figure 2C). Furthermore, DIF-1 can promote cellular glucose uptake in the presence or absence of insulin and insulin receptors in vitro [14,15,16]. These observations suggest that DIF-1 is a promising lead for the development of orally administrable antiobesity and antidiabetic drugs, which may be effective hopefully for both type 1 and type 2 diabetes.

### 3.2. Mechanism of the Action of DIF-1 to Promote Glucose Uptake: Pharmacological Target of DIF-1

The pharmacological target molecules of DIF-1 and DIF-3 that mediate their growth-suppressive and glucose-metabolizing effects in mammalian cells have not yet been fully identified. We have previously shown with commercially available bovine PDE1 and calmodulin that DIF-1 and DIF-3 inhibit PDE1 activity (*K*_i_ value of DIF-1: 4.5–5 μM) by competing with the PDE1 substrate cAMP in vitro, which suggests that DIF-1 and DIF-3 at 10–40 μM may suppress tumor cell growth, at least in part by directly inhibiting PDE1 [16,19]. However, since the PDE1 inhibitor, 8-methoxymethyl-3-isobutyl-1-methylxanthine, has limited antiproliferative effects on tumor cells [16,19], the antiproliferative effect of DIFs through PDE1 inhibition is likely modest, if any. Alternatively, since DIFs have biological activities other than those mentioned above [11], PDE1 may be involved in these biological activities.

Further, using affinity chromatography and LC–MS/MS, Matsuda et al. [23] identified MDH2 as a DBP in HeLa cells. MDH2 is one of the essential enzymes in the tricarboxylic acid cycle, which oxidizes malate to oxaloacetate in the cycle, and is involved in malate-aspartate shuttle across the mitochondrial membrane for energy production in all eukaryotic cells [45,46,47]. It was further shown with commercially available porcine MDH2 that DIF-1 bound MDH2 and inhibited its activity in vitro, whereas DIF-3 did not significantly inhibit MDH2 in vitro [23], although DIF-3 had stronger growth-inhibitory activity than DIF-1 [5,11] (Figure 2A,B) (Table 1). Therefore, the overall role of MDH2 inhibition as part of DIF-1 actions in mammalian cells remained unclear.

In the present study, by performing affinity chromatography with DIF beads (Figure 1B) different from those used by Matsuda et al. [23] and using LC–MS/MS, we again identified MDH2 as a DBP in both HeLa and 3T3-L1 cells (Figure 3). To elucidate the involvement of MDH2 in the growth of HeLa and 3T3-L1 cells and glucose uptake in confluent 3T3-L1 cells, we examined the effects of the MDH2 inhibitor LW6 on the phenomena. LW6 at 2–10 μM did not significantly suppress the growth of HeLa cells, whereas LW6 at 20 μM significantly suppressed it (Figure 4B,D). However, LW6 at 2–20 μM significantly promoted glucose uptake in confluent 3T3-L1 cells, with 10 μM LW6 showing the strongest activity (Figure 4F). These results suggest that MDH2 inhibition suppressed cell growth and promoted glucose uptake in these cells, but MDH2 inhibition appeared to promote glucose uptake more strongly than it suppressed cell growth (Table 1).

However, LW6 is known to inhibit not only MDH2 but also HIF-1α (hypoxia-inducible factor 1α) [25,48]; LW6 is thought to suppress the growth of some tumor cells by inducing HIF-1α degradation, at least in part [48,49]. However, it is unlikely that LW6 promotes glucose uptake via HIF-1α inhibition, because HIF-1α promotes glucose uptake in many tumor cells by increasing GLUT1 expression [50,51]. Therefore, LW6 (10 μM) could have promoted glucose uptake via MDH2 inhibition rather than HIF-1α inhibition in confluent 3T3-L1 cells (Figure 4F and Figure 5A,B). Other research groups have shown that LW6 at 30–80 μM significantly suppressed cell proliferation in human activated T cells [26] and rat pancreatic adenocarcinoma cells [52], that LW6 at 15 μM inhibited MDH2 activity in the lysates of human renal proximal tubular epithelial cells by 30%, and that LW6 at 30–60 μM inhibited it by ~80% [28]. Taken together, these findings suggest that weak and moderate inhibition of MDH2 may induce glucose uptake, whereas strong inhibition of MDH2 and HIF-1α may induce growth suppression in these cells.

We have previously shown that DIF-1 and DIF-3 function as mitochondrial uncouplers and that DIFs may exert their antitumor effects partly through this function [6,8]. It has been further shown that DIF-1 promotes glucose uptake, at least in part, by uncoupling mitochondria and increasing intracellular cAMP levels [15,16]. In the present study, an examination of the combinatorial effects of LW6, DNP, and Br-cAMP (Figure 5A) suggested that DIF-1 promoted glucose uptake by inhibiting MDH2, uncoupling mitochondria, and increasing intracellular cAMP levels in 3T3-L1 cells (Figure 5B).

Other studies have shown that LW6 at 10 μM decreased cellular ATP content and activated AMPK in human colorectal carcinoma HCT116 cells [25], that DNP at 0.5 mM also decreased cellular ATP content, increased AMPK activity, and promoted glucose uptake in rat L6 skeletal muscle cells [53], and that 0.1 mM DNP showed similar effects in 3T3-L1 cells [15]. Given that LW6 (10 μM) and/or DNP (0.1 mM) significantly activated AMPK and promoted glucose uptake in 3T3-L1 cells (Figure 5A,D) [15], MDH2 inhibition and mitochondrial uncoupling appeared to cooperate to reduce ATP concentration, increase AMPK activity, and promote glucose uptake (Figure 5B).

## 4. Materials and Methods

### 4.1. Cells and Reagents

Mouse 3T3-L1 fibroblasts and human cervical cancer HeLa cells were used. Cells were maintained at 37 °C (5% CO_2_) in Dulbecco’s Modified Eagle’s Medium (DMEM) containing a high concentration (4.5 mg/mL) of glucose (Fujifilm Wako Pure Chemical Corporation, Osaka, Japan) supplemented with 75 μg/mL penicillin, 50 μg/mL streptomycin, and 10% (*v*/*v*) heat-inactivated fetal bovine serum, referred to as DMEM-HG. DIF-1, DIF-3, THPH, and DIF-1-NH_2_ were synthesized as previously described [5,18]. DIF-1 and DIF-3 were dissolved in dimethylsulfoxide (DMSO) at 5–20 mM, while THPH was dissolved in ethanol (EtOH), and stored at −20 °C. LW6 was obtained from Sellec Chemicals (Houston, TX, USA). DNP and AICAR were obtained from Fujifilm Wako Pure Chemical Corporation, and Br-cAMP was from Sigma (St. Louis, MO, USA). DMSO solutions of DNP (50–100 mM) and LW6 (2–20 mM) and an aqueous solution of Br-cAMP (100 mM) were stored at −20 °C. Rabbit antibodies against AMPKα, phospho-AMPKα, and glyceraldehyde 3-phosphate dehydrogenase (GAPDH) were purchased from Cell Signaling Technology (Beverly, MA, USA), and alkaline phosphatase-conjugated goat anti-rabbit IgG antibody was purchased from Promega (Madison, WI, USA).

### 4.2. Assessment of Glucose Consumption (Uptake) in 3T3-L1 Cells

The rate of glucose consumption was assessed mostly as previously described [14,15,16]. The 3T3-L1 cells were incubated in DMEM-HG (1 mL per well) for 3–5 days in a 12-well plate (Corning, New York, NY, USA) until they reached confluency. The cells were then incubated for 16–20 h with the additives in 1 mL of fresh DMEM containing a medium concentration (2 mg/mL) of glucose, 10% fetal bovine serum, the antibiotics, and 10 mM HEPES-NaOH (pH 7.4), referred to as DMEM-MG. Glucose concentration in DMEM-MG in each well was measured using a handheld glucose monitor (GlucCell) and its sensor chips (Glucose Test Strips), both obtained from Cesco Bioengineering Co., Ltd. (Taichung, Taiwan), and the rate of glucose consumption was calculated. Here, glucose consumption was taken as a measure of glucose uptake, as explained previously [14,15,16].

### 4.3. Cell Growth Assay

Cells (3T3-L1 or HeLa) were incubated in 12-well plates in DMEM-HG (5 × 10^3^ cells in 1 mL per well) overnight until they adhered to the bottom. The medium was replaced with 1 mL of fresh DMEM-HG containing additives, and the cells were grown for 3 days. The media were removed, and the cells were washed with 0.5 mL of phosphate-buffered saline (pH 7.4) without Ca^2+^ and Mg^2+^ (PBS(–)) and incubated in 0.5 mL of DMEM-HG without phenol red (Fujifilm Wako Pure Chemical Corporation) containing 5% (*v*/*v*) Alamar Blue (Fujifilm Wako Pure Chemical Corporation), a cell number indicator [5,54], until the color of the media changed from blue to reddish purple. The cell number relative to the control was determined by measuring the absorbance at 570 nm (reference at 595 nm).

### 4.4. Coupling of DIF-1-NH_2_ and Affi-Gel 10 Resin

DIF-1-NH_2_ was coupled to Affi-Gel 10 resin (Bio-Rad, Hercules, CA, USA) at 4 °C to produce DIF–Affi-Gel 10 (DIF beads) (Figure 1B) according to the manufacturer’s instructions, as previously described [18]. The DIF beads were washed well with 0.1 M phosphate-buffered saline and kept in EtOH at 4 °C until use.

### 4.5. Identification of DBPs in HeLa and 3T3-L1 Cells

#### 4.5.1. Preparation of Cell Extracts for Affinity Chromatography

HeLa cells or 3T3-L1 cells were cultured in 12-well plates in DMEM-HG (1 mL per well) until 90–95% (HeLa) or 100% (3T3-L1) confluent. The cells in four wells were then washed each with 1 mL of PBS(–) four times, and the cell proteins in four wells were collected sequentially by adding 1.2 mL of 10 mM Tris-HCl pH 7.4, 150 mM NaCl, 1 mM EDTA, 0.05% Tween 20 (TBS-T) and the recommended amounts (in the manufacturer’s instructions) of Complete Protease Inhibitor Cocktail (Roche Diagnostics, Mannheim, Germany). The cell lysates were placed in 1.5 mL microcentrifuge tubes and spun (12,000× *g* for 10 min at 4 °C) to remove insoluble cell debris. Then 1/200 volume of 20 mM THPH was added to the resulting supernatant (cell extract) to a final concentration of 0.1 mM, which was used for affinity purification of DBPs.

#### 4.5.2. Affinity Chromatography and SDS-PAGE

Affi-Gel 10 resin (control beads) and DIF beads, each at a volume of about 50 μL, were washed well (equilibrated) with TBS-T in 1.5 mL microcentrifuge tubes and then several times with TBS-T containing 0.1 mM THPH. THPH was added to block the nonspecific binding of cell proteins to the beads. Then, 600 μL of each cell extract was added to tubes containing control beads or DIF beads, which were rotated gently for 2 h on a microcentrifuge tube rotator (MTR-103, As One Corporation, Osaka, Japan) at 4 °C. The beads were then pelleted, the supernatants were removed, and the beads were washed five times with 0.5 mL of TBS-T containing 0.1 mM THPH. The proteins bound to the control beads and DIF beads were collected by directly adding 50 μL of SDS sample buffer, comprising 70 mM Tris-HCl pH 6.8, 2% (*w*/*v*) SDS, 2.5% (*v*/*v*) 2-mercaptoethanol, 10% (*w*/*v*) glycerol, 0.01% (*w*/*v*) bromophenol blue, and the Complete Protease Inhibitor Cocktail, to the beads.

The collected proteins were separated by SDS-PAGE (10% gel) and stained using a silver staining kit (2D-Silver Stain-2; Cosmo Bio Co., Ltd., Tokyo, Japan). The bands of proteins specifically bound to DIF beads were cut off with a cutter, and each band was cut into three pieces and placed into a 1.5 mL microcentrifuge tube. The silver-stained gel pieces were destained by washing them several times with 1 mL of an aqueous destaining solution (1.85 mg/mL NaCl, 1.85 mg/mL CuSO_4_, 21.8 mg/mL Na_2_S_2_O_3_, and a very small and appropriate amount of NH_4_OH) according to the manufacturer’s instructions (Cosmo Bio Co., Ltd.). The gel pieces were dehydrated by washing them three times with 1 mL of H_2_O and three times with 0.5 mL of acetonitrile, and finally air-drying them.

#### 4.5.3. LC–MS/MS

The gel pieces were treated as described previously [55,56]. After digestion with trypsin, the resulting peptides were extracted from the gel pieces three times with 0.1% formic acid in 50% acetonitrile, combined, desalted using a styrene divinylbenzene tip (GL Science, Tokyo, Japan), and lyophilized using a centrifugal evaporator. The peptides were identified by nano LC–MS/MS using the Triple TOF 5600+ System operated with Analyst TF 1.7 software and an Eksigent nano LC system (SCIEX, Framingham, MA, USA). The MS data obtained were analyzed using ProteinPilot 5.0.2 Software (SCIEX) and the UniProt database (2020_04). A confidence cutoff of a 1% false discovery rate was applied for protein identification.

### 4.6. Western Blotting

The 3T3-L1 cells (in a 12-well plate) were washed with 1 mL/well of PBS, harvested, and lysed by adding an SDS-sample buffer solution (150–200 μL/well), destroyed and heated by sonication, and used for SDS-PAGE. Protein transfer and immunoblotting were performed as described previously [15], using primary rabbit antibodies for AMPKα, phospho-AMPKα, or GAPDH, and a secondary antibody, alkaline-phosphatase-conjugated goat anti-rabbit IgG antibody. Visualized protein bands were then digitized and quantified by using Adobe Photoshop CS4 (version 11.0) (Adobe, San Jose, CA, USA) and ImageJ software (version 1.53a) (http://imagej.nih.gov/ij/) (accessed on 30 March 2023).

### 4.7. Statistical Analyses

Statistical analyses were performed by Student’s *t*-test (two-tailed, unpaired) or one-way analysis of variance (ANOVA), followed by Tukey’s multiple comparison test. Values were considered to be significantly different when the *p* value was less than 0.05.

## 5. Conclusions

In the present study, to elucidate the mechanism(s) underlying the glucose-uptake-promoting effect of DIF-1, we tried to identify the pharmacological target molecules of DIF-1 in 3T3-L1 fibroblasts, using HeLa cells as a reference. Here, we showed that DIF-1 promoted glucose uptake, at least in part via direct inhibition of MDH2 in these cells, thus presenting MDH2 as a potential pharmacological target molecule of DIF-1 for promoting glucose uptake. Furthermore, we showed that DIF-1 might promote glucose uptake, at least in part, by inhibiting MDH2, uncoupling mitochondria, and subsequently activating AMPK in 3T3-L1 cells. Some of the antidiabetes drugs currently in therapeutic use may have functions that have not yet been elucidated, but so far, there are no drugs that have the same mechanism of action as DIF-1.

## Figures and Tables

**Table 1:** Comparison of the biological activities of DIF-1, DIF-3, and LW6.

Compound (Concentration)	Growth Inhibition *	Glucose-Uptake Promotion *	MDH2 Inhibition
DIF-1 (20 μM)	±	++	+ **
DIF-3 (20 μM)	+	±	– **
LW6 (10 μM)	–	+	++ ***

Footnotes: * The effects of DIFs at 20 μM and LW6 at 10 μM on cell growth and glucose uptake in 3T3-L1 cells (Figure 2 and Figure 4) are shown. Growth inhibition: +, ~50% inhibition; ±, slight (or not significant) inhibition; –, no significant inhibition. Glucose-uptake promotion: ++, ≥2-fold promotion; +, 1.5-fold promotion; ±, no significant promotion. The effects of ** DIFs [23] and *** LW6 [25] on MDH2 activity in vitro are shown. MDH2 inhibition: ++, ≥50% inhibition; +, ~40% inhibition; –, no significant inhibition.

## Data Availability

The data presented in this study are available in this article.

## References

[1] Morris H.R., Taylor G.W., Masento M.S., Jermyn K.A., Kay R.R. (1987). Chemical structure of the morphogen differentiation inducing factor from *Dictyostelium discoideum*. Nature.

[2] Morris H.R., Masento M.S., Taylor G.W., Jermyn K.A., Kay R.R. (1988). Structure elucidation of two differentiation inducing factors (DIF-2 and DIF-3) from the cellular slime mould *Dictyostelium discoideum*. Biochem. J..

[3] Kay R.R., Berks M., Traynor D. (1989). Morphogen hunting in *Dictyostelium discoideum*. Development.

[4] Kay R.R., Flatman P., Thompson C.R.L. (1999). DIF signalling and cell fate. Semin. Cell Develop. Biol..

[5] Gokan N., Kikuchi H., Nakamura K., Oshima Y., Hosaka K., Kubohara Y. (2005). Structural requirements of *Dictyostelium* differentiation-inducing factors for their stalk-cell-inducing activity in *Dictyostelium* cells and anti-proliferative activity in K562 human leukemic cells. Biochem. Pharmacol..

[6] Kubohara Y., Kikuchi H., Matsuo Y., Oshima Y., Homma Y. (2013). Mitochondria are the target organelle of differentiation-inducing factor-3, an anti-tumor agent isolated from *Dictyostelium discoideum*. PLoS ONE.

[7] Takahashi-Yanaga F., Yoshihara T., Jingushi K., Igawa K., Tomooka K., Watanabe Y., Morimoto S., Nakatsu Y., Tsuzuki T., Nakabeppu Y. (2014). DIF-1 inhibits tumor growth *in vivo* reducing phosphorylation of GSK-3β and expressions of cyclin D1 and TCF7L2 in cancer model mice. Biochem. Pharmacol..

[8] Kubohara Y., Komachi M., Homma Y., Kikuchi H., Oshima Y. (2015). Derivatives of *Dictyostelium* differentiation-inducing factors inhibit lysophosphatidic acid–stimulated migration of murine osteosarcoma LM8 cells. Biochem. Biophys. Res. Commun..

[9] Arioka M., Takahashi-Yanaga F., Kubo M., Igawa K., Tomooka K., Sasaguri T. (2017). Anti-tumor effects of differentiation-inducing factor-1 in malignant melanoma: GSK-3-mediated inhibition of cell proliferation and GSK-3-independent suppression of cell migration and invasion. Biochem. Pharmacol..

[10] Arioka M., Seto-Tetsuo F., Inoue T., Miura K., Ishikane S., Igawa K., Tomooka K., Takahashi-Yanaga F., Sasaguri T. (2023). Differentiation-inducing factor-1 reduces lipopolysaccharide-induced vascular cell adhesion molecule-1 by suppressing mTORC1-S6K signaling in vascular endothelial cells. Life Sci..

[11] Kubohara Y., Kikuchi H. (2019). *Dictyostelium*: An important source of structural and functional diversity in drug discovery. Cells.

[12] Seto-Tetsuo F., Arioka M., Miura K., Inoue T., Igawa K., Tomooka K., Takahashi-Yanaga F., Sasaguri T. (2021). DIF-1 inhibits growth and metastasis of triple-negative breast cancer through AMPK-mediated inhibition of the mTORC1-S6K signaling pathway. Oncogene.

[13] Seto-Tetsuo F., Arioka M., Miura K., Inoue T., Igawa K., Tomooka K., Sasaguri T. (2023). DIF-1 exhibits anticancer activity in breast cancer via inhibition of CXCLs/CXCR2 axis-mediated communication between cancer-associated fibroblasts and cancer cells. Int. Immunopharmacol..

[14] Omata W., Shibata H., Nagasawa M., Kojima I., Kikuchi H., Oshima Y., Hosaka K., Kubohara Y. (2007). *Dictyostelium* differentiation-inducing factor-1 induces glucose transporter 1 translocation and promotes glucose uptake in mammalian cells. FEBS J..

[15] Kubohara Y., Homma Y., Shibata H., Oshima Y., Kikuchi H. (2021). *Dictyostelium* differentiation-inducing factor-1 promotes glucose uptake, at least in part, via an AMPK-dependent pathway in mouse 3T3-L1 cells. Int. J. Mol. Sci..

[16] Kubohara Y., Fukunaga Y., Kikuchi H., Kuwayama H. (2023). Pharmacological evidence that *Dictyostelium* differentiation-inducing factor 1 promotes glucose uptake partly via an increase in intracellular cAMP content in mouse 3T3-L1 cells. Molecules.

[17] Kawaharada R., Nakamura A., Takahashi K., Kikuchi H., Oshima Y., Kubohara Y. (2016). Oral administration of *Dictyostelium* differentiation-inducing factor 1 lowers blood glucose levels in streptozotocin-induced diabetic rats. Life Sci..

[18] Kubohara Y., Kikuchi H., Nakamura K., Matsuo Y., Oshima Y. (2010). Preparation of an antibody that recognizes and neutralizes Dictyostelium differentiation-inducing factor-1. Biochem. Biophys. Res. Commun..

[19] Shimizu K., Murata T., Tagawa T., Takahashi K., Ishikawa R., Abe Y., Hosaka K., Kubohara Y. (2004). Calmodulin-dependent cyclic nucleotide phosphodiesterase (PDE1) is a pharmacological target of differentiation-inducing factor-1, an anti-tumor agent isolated from *Dictyostelium*. Cancer Res..

[20] Dousa T.P. (1999). Cyclic-3′,5′-nucleotide phosphodiesterase isozymes in cell biology and pathophysiology of the kidney. Kidney Int..

[21] Kakkar R., Raju R.V., Sharma R.K. (1999). Calmodulin-dependent cyclic nucleotide phosphodiesterase (PDE1). Cell Mol. Life Sci..

[22] Samidurai A., Xi L., Das A., Iness A.N., Vigneshwar N.G., Li P.L., Singla D.K., Muniyan S., Batra S.K., Kukreja R.C. (2021). Role of phosphodiesterase 1 in the pathophysiology of diseases and potential therapeutic opportunities. Pharmacol. Ther..

[23] Matsuda T., Takahashi-Yanaga F., Yoshihara T., Maenaka K., Watanabe Y., Miwa Y., Morimoto S., Kubohara Y., Hirata M., Sasaguri T. (2010). *Dictyostelium* differentiation-inducing factor-1 binds to mitochondrial malate dehydrogenase and inhibits its activity. J. Pharmacol. Sci..

[24] Lee K., Ban H.S., Naik R., Hong Y.S., Son S., Kim B.K., Xia Y., Song K.B., Lee H.S., Won M. (2013). Identification of malate dehydrogenase 2 as a target protein of the HIF-1 inhibitor LW6 using chemical probes. Angew. Chem. Int. Ed. Engl..

[25] Naik R., Won M., Ban H.S., Bhattarai D., Xu X., Eo Y., Hong Y.S., Singh S., Choi Y., Ahn H.C. (2014). Synthesis and structure-activity relationship study of chemical probes as hypoxia induced factor-1α/malate dehydrogenase 2 inhibitors. J. Med. Chem..

[26] Eleftheriadis T., Pissas G., Antoniadi G., Liakopoulos V., Stefanidis I. (2015). Malate dehydrogenase-2 inhibitor LW6 promotes metabolic adaptations and reduces proliferation and apoptosis in activated human T-cells. Exp. Ther. Med..

[27] Eleftheriadis T., Pissas G., Mavropoulos A., Liakopoulos V., Stefanidis I. (2017). Comparison of the effect of the aerobic glycolysis inhibitor dichloroacetate and of the Krebs cycle inhibitor LW6 on cellular and humoral alloimmunity. Biomed. Rep..

[28] Eleftheriadis T., Pissas G., Golfinopoulos S., Efthymiadi M., Liakopoulos V., Stefanidis I. (2022). Inhibition of malate dehydrogenase-2 protects renal tubular epithelial cells from anoxia-reoxygenation-induced death or senescence. Biomolecules.

[29] Corton J.M., Gillespie J.G., Hardie D.G. (1994). Role of the AMP-activated protein kinase in the cellular stress response. Curr. Biol..

[30] Abbud W., Habinowski S., Zhang J.Z., Kendrew J., Elkairi F.S., Kemp B.E., Witters L.A., Ismail-Beigi F. (2000). Stimulation of AMP-activated protein kinase (AMPK) is associated with enhancement of Glut1-mediated glucose transport. Arch. Biochem. Biophys..

[31] Barnes K., Ingram J.C., Porras O.H., Barros L.F., Hudson E.R., Fryer L.G., Foufelle F., Carling D., Hardie D.G., Baldwin S.A. (2002). Activation of GLUT1 by metabolic and osmotic stress: Potential involvement of AMP-activated protein kinase (AMPK). J. Cell Sci..

[32] Woods A., Johnstone S.R., Dickerson K., Leiper F.C., Fryer L.G., Neumann D., Schlattner U., Wallimann T., Carlson M., Carling D. (2003). LKB1 is the upstream kinase in the AMP-activated protein kinase cascade. Curr. Biol..

[33] Sullivan J.E., Brocklehurst K.J., Marley A.E., Carey F., Carling D., Beri R.K. (1994). Inhibition of lipolysis and lipogenesis in isolated rat adipocytes with AICAR, a cell-permeable activator of AMP-activated protein kinase. FEBS Lett..

[34] Kim J., Yang G., Kim Y., Kim J., Ha J. (2016). AMPK activators: Mechanisms of action and physiological activities. Exp. Mol. Med..

[35] Kerner W., Brückel J.J.E. (2014). Definition, classification and diagnosis of diabetes mellitus. Clin. Endocrinol. Diabetes.

[36] Melmer A., Laimer M.J.N. (2016). Treatment goals in diabetes. Nov. Diabetes.

[37] Chen Q., Zhu L., Tang Y., Zhao Z., Yi T., Chen H.J.A. (2017). Preparation-related structural diversity and medical potential in the treatment of diabetes mellitus with ginseng pectins. Ann. N. Y. Acad. Sci..

[38] Guo Y., Baldelli A., Singh A., Fathordoobady F., Kitts D., Pratap-Singh A. (2022). Production of high loading insulin nanoparticles suitable for oral delivery by spray drying and freeze drying techniques. Sci. Rep..

[39] Mootoosamy A., Mahomoodally M.F. (2014). Ethnomedicinal application of native remedies used against diabetes and related complications in mauritius. J. Ethnopharmacol..

[40] Weinberg Sibony R., Segev O., Dor S., Raz I. (2023). Drug Therapies for diabetes. Int. J. Mol. Sci..

[41] Chong K., Chang J.K.-j., Chuang L.-M. (2024). Recent advances in the treatment of type 2 diabetes mellitus using new drug therapies. Kaohsiung J. Med. Sci..

[42] Yang M.-H., Yang Y., Zhou X., Chen H.-G. (2024). Advances in polysaccharides of natural source of anti-diabetes effect and mechanism. Mol. Biol. Rep..

[43] Wurster B., Kay R.R. (1990). New roles for DIF? Effects on early development in *Dictyostelium*. Dev. Biol..

[44] Morandini P., Offer J., Traynor D., Nayler O., Neuhaus D., Taylor G.W., Kay R.R. (1995). The proximal pathway of metabolism of the chlorinated signal molecule differentiation-inducing factor-1 (DIF-1) in the cellular slime mould *Dictyostelium*. Biochem. J..

[45] Gietl C. (1992). Malate dehydrogenase isoenzymes: Cellular locations and role in the flow of metabolites between the cytoplasm and cell organelles. Biochim. Biophys. Acta.

[46] Goward C.R., Nicholls D.J. (1994). Malate dehydrogenase: A model for structure, evolution, and catalysis. Protein Sci..

[47] Minárik P., Tomásková N., Kollárová M., Antalík M. (2002). Malate dehydrogenases—Structure and function. Gen. Physiol. Biophys..

[48] Lee K., Kang J.E., Park S.K., Jin Y., Chung K.S., Kim H.M., Lee K., Kang M.R., Lee M.K., Song K.B. (2010). LW6, a novel HIF-1 inhibitor, promotes proteasomal degradation of HIF-1alpha via upregulation of VHL in a colon cancer cell line. Biochem. Pharmacol..

[49] Xu H., Chen Y., Li Z., Zhang H., Liu J., Han J. (2022). The hypoxia-inducible factor 1 inhibitor LW6 mediates the HIF-1α/PD-L1 axis and suppresses tumor growth of hepatocellular carcinoma in vitro and in vivo. Eur. J. Pharmacol..

[50] Brahimi-Horn M.C., Chiche J., Pouysségur J. (2007). Hypoxia signalling controls metabolic demand. Curr. Opin. Cell Biol..

[51] Heydarzadeh S., Moshtaghie A.A., Daneshpoor M., Hedayati M. (2020). Regulators of glucose uptake in thyroid cancer cell lines. Cell Commun. Signal..

[52] Zhang X., Liu P., Shang Y., Kerndl H., Kumstel S., Gong P., Vollmar B., Zechner D. (2020). Metformin and LW6 impairs pancreatic cancer cells and reduces nuclear localization of YAP1. J. Cancer.

[53] Patel N., Khayat Z.A., Ruderman N.B., Klip A. (2001). Dissociation of 5’ AMP-activated protein kinase activation and glucose uptake stimulation by mitochondrial uncoupling and hyperosmolar stress: Differential sensitivities to intracellular Ca^2+^ and protein kinase C inhibition. Biochem. Biophys. Res. Commun..

[54] Ahmed S.A., Gogal R.M., Walsh J.E. (1994). A new rapid and simple non-radioactive assay to monitor and determine the proliferation of lymphocytes: An alternative to [3H]thymidine incorporation assay. J. Immunol. Methods.

[55] Goodman J.K., Zampronio C.G., Jones A.M.E. (2018). Hernandez-Fernaud, J.R. Updates of the in-gel digestion method for protein analysis by mass spectrometry. Proteomics.

[56] Nihei Y., Haniuda K., Higashiyama M., Asami S., Iwasaki H., Fukao Y., Nakayama M., Suzuki H., Kikkawa M., Kazuno S. (2023). Identification of IgA autoantibodies targeting mesangial cells redefines the pathogenesis of IgA nephropathy. Sci. Adv..

